# Cultural influence on the expression of labour-associated pain

**DOI:** 10.1186/s12884-022-05173-1

**Published:** 2022-11-14

**Authors:** S Navarro-Prado, MA Sánchez-Ojeda, J Marmolejo-Martín, G Kapravelou, E Fernández-Gómez, A Martín-Salvador

**Affiliations:** 1 Department of Nursing, Faculty of Health Sciences, University of Granada, 52071 Melilla, Spain; 2 Department of Statistics and Operations Research, Faculty of Social and Legal Sciences, University of Granada, 52071 Melilla, Spain; 3 Department of Physiology, Faculty of Health Sciences, University of Granada, 52071 Melilla, Spain

**Keywords:** Labour pain, Culture, Communication barriers, Midwife care, Support

## Abstract

**Background:**

Every woman expresses pain differently during birth since it depends on a multitude of predictive factors. The medical care received, companionship during birth, cultural background and language barriers of the women in labour can influence on the expression of pain. This study aims to evaluate the expression of pain during birth and its associated factors in women treated in a Spanish border town.

**Methods:**

The study included 246 women in labour. The expression of pain during labour was evaluated using the validated ESVADOPA scale. A descriptive analysis and association study were performed between cultural identity and dimensions of the scale. Multiple linear regression models were performed to assess the association between cultural identity, origin, language barrier, and companionship during labour.

**Results:**

The women included in the study comprised 68.7% Berbers, 71.5% Muslims and 82.1% were accompanied during labour. An association between cultural identity and greater body expression of pain (*p* = 0.020; Cramer’s V = 0.163) in addition to its verbal expression was found during the latent phase of labour, (*p* = 0.028; Cramer’s V = 0.159). During the active phase of labour, cultural identity was associated with pain expression through greater body response, verbal expression, expression of the facial muscles, anxiety, inability to relax and vegetative symptoms. The different factors studied that had a predictive value were companionship (*p* = 0.027) during the latent phase of labour and Berber origin (*p* = 0.000), language barrier (*p* = 0.014) and companionship (*p* = 0.005) during the active phase of labour. The models designed predict pain expression in the latent phase by companionship and type of companionship (β = 1.483; 95%CI = 0.459–2.506, β = 0.238; 95%CI = 0.029–0. 448, respectively), and in the active phase by background, language barrier and companionship (β = 0.728; 95%CI = 0.258–1.198, β = 0.738; 95%CI = 0.150–1.326, β = 1.888; 95%CI = 0.984–2.791, respectively).

**Conclusion:**

Culture, origin, language barrier and companionship during labour influences the manner in which women in labour express their pain. An understanding of this may help midwives correctly interpret the signs of pain expression and be able to offer the appropriate assistance depending on a woman's particular characteristics. There is a clear need for new models of maternity care that will take the cultural and language characteristics of women in labour into consideration.

**Supplementary Information:**

The online version contains supplementary material available at 10.1186/s12884-022-05173-1.

## Background

How pain is experienced during labour is unique for every woman and begins with the appearance of the first contractions caused by lightening and the exit of the foetus and the increased pressure placed on the adnexa of the uterus [1, 2].

The expression of labour pain is influenced by internal factors such as previous experiences of pain, the concept of pregnancy and birth created, the degree of fear and anxiety that the process causes, obstetric history, emotional and physical conditions, as well as the woman’s expectations of childbirth. They can all be modulated from external factors such as the healthcare received, family support during pregnancy, companionship during labour, established cultural patterns and knowledge of pain-relief methods during labour [3, 4].

The combination of these internal and external factors may determine how women in labour express the pain they feel throughout the process. Furthermore, other factors, such as the foetal position during lightening and the nature of the contractions, may also influence the expression of pain during labour. Occasionally, this kind of acquired behaviour may compromise the mother’s as well as the baby's health since this altered state may increase the release of catecholamines and cause labour dystocia and foetal distress [2, 5].

Hence, it is well known that labour pain is a major concern for pregnant women and their partners [6], especially for those about to have their first baby [7, 8]. Unlike their partners who are largely concerned about the pain that women in labour may feel, young mothers are also worried about the information that they receive on how to cope with the entire process, whether or not they will be supported in addition to the care that the new-born will be offered [3].

This information is offered to pregnant women during antenatal screenings as part of prenatal care and maternal education sessions during which they are also informed about their right to be accompanied by a person of their choice throughout the process [9]. During the preparation sessions, not only pregnant women but also those who will accompany them during labour are instructed to ensure the effectiveness of the process [10].

Even though Western countries have greater and better availability of maternity healthcare services than those of developing countries, it has been observed that migrant women have to overcome numerous problems in order access them, and, therefore, only request health-care at the time of the labour [11]. Beliefs or expectations, established cultural practices and negative attitudes of the health care personnel towards cultural differences and language barriers are among the motives that can explain the less demanding attitude of migrant women, [1, 12, 13]. Nonetheless, some healthcare centres have interpreters to assist communication between pregnant women and midwives. However, this service is often not requested since it eliminates the women’s privacy [14–16].

In order to be able to offer high- quality care and information, healthcare professionals should be trained to deal with the multi-cultural perspective of today’s society [17, 18]. According to the Spanish National Statistics Institute [19], 359,770 births were registered in Spain in 2019, 22.3% of which were births by migrant women [20]. This migration data is similar to the majority of countries of the European Union.

The Autonomous city of Melilla is a Spanish city situated in North Africa with the particular characteristics of a typical border city. It had a population of 86,487 in 2019 [21] to which a total of 13,363 principally Moroccan immigrants were added. However, the most significant fact is that around 30,000 Moroccans enter the city to work, do business, visit or even seek medical care [22] on a daily basis. Women make up over 50% of the total population, with a higher fertility rate compared to the Spanish national average: 42.70 among the population registered in the census and 203.60 among the immigrant population [21].

This data shows that there is a very high demand placed on maternity services, which must adapt to the characteristics of each woman [12, 16, 18]. In this regard, the care that midwives offer during labour should be individualised and focused on the specific characteristics of each woman. This will allay fears concerning labour and achieve a better way of coping with the labour-related pain [6, 23].

Culture is defined as the body of knowledge, ideas, traditions and customs that characterise a given community, and the expression of pain in childbirth is a socio-cultural construct. It is observed that, depending on the culture of the woman in labour, there are women who endure contractions with minimal facial or bodily expressions, and in contrast, other women face this pain with exacerbated verbal expressions. Furthermore, culture also seems to be a determining factor in terms of the modesty of being treated by men, accepting epidural analgesia or the need for accompaniment during labour [8, 11].

Hence, the aim of this research is to assess the expression of labour pain who are treated in a border city hospital and to discover the associated factors that may affect the expression of this pain.

## Methods

### Sample

This research was carried out in Melilla, a border city with Morocco. The sample selection was performed in the last quarter of 2019, making up 41.14% of the births that took place in the city hospital The sample was initially formed by 326 women in labour and was reduced to 246 women, after the application of inclusion and exclusion criteria. The final sample was made up of pregnancies with a single foetus, without the administration of epidural anaesthesia or which had been administered during the active phase of labour, thus excluding the epidurals were administered in early stages of the process.

### Instruments

To evaluate the response of women's labour pain, the Rating Scale of Pain during Childbirth (ESVADOPA) was used, created and validated previously by Navarro-Prado et al. [4]. This scale evaluates the response to labour-associated pain using five items: facial muscles, body response, verbal response, restlessness, ability to relax and vegetative symptoms. Each item is evaluated using a scale from 0 to 3, and the maximum pain expression of the woman in labour is taken into account. The sum of all the items is used to classify the expression of pain during labour in five categories: < 1: Does not express pain; 1–6: Expresses mild pain; 7–12: Expresses moderate pain; and 13–18: Expresses intense pain (Annex 1).

A great advantage of this scale is that it can be completed by the observations of pain expression during the contractions by the midwife assisting the labour, and it therefore does not interfere with the labour process or involve the intervention of the woman in labour. In this regard, it preserves the intimacy of the labour process and means it is not necessary that the midwives and women in labour share a common language, an important factor since the research was performed in a border city where the language barrier is a considerable obstacle for the healthcare services.

### Procedure

After the women in labour were included in the research, they completed a questionnaire regarding socio-demographic variables and obstetric history. Specifically, these variables included:

- Socio-demographic variables: age, ethnic origin (European, Berber, Sub-Saharan, Latin American), existence of language barriers (yes/no), cultural identity (Christian/ Muslim/ Jew/ atheist). For cultural identity, participants were asked to self-identify with one of the previous categories that their lifestyle and habits most fitted into.
- Obstetric variables: companionship during labour (yes/no), relationship to the companion (partner/ mother/ sister/ mother-in-law/ friend/ other female family member), use of oxytocin (yes/no).

To ensure the completion of the questionnaire, help was provided to women with a language barrier by healthcare professionals who knew their maternal language.

Once admitted to the delivery service, the expression of pain was evaluated twice in each pregnant woman. The first evaluation was carried out during the latent phase of labour with a cervical dilation of 2–4 cm. The midwife observed the woman’s reaction to the pain caused by a contraction and, after carrying out the assessment, she filled in the ESVADOPA scale obtaining a total score.

The second evaluation was carried out during the active phase of labour with a cervical dilation of 6–7 cm. This assessment was carried out prior to the start of the pushing to ensure that there was no confusion in the expression of pain due to the body response produced by pushing. This second evaluation was only carried out in women who had not received epidural anaesthesia or whose administration was received after reaching 6–7 cm of dilation. This fact greatly decreased the available sample since many women opted for epidural anaesthesia in the early stages of labour. All scales were completed by the same two midwives who assisted in the labour of the participants.

### Data analysis

A descriptive, analytical and cross-sectional study was carried out. Thanks to this initial analysis, we have obtained, among other things, measures of central tendency and frequencies of each parameter studied. Subsequently, an association analysis (contingency tables) was performed between the cultural identity of the women (independent variable) and the scores of the different parameters of the scale as well as the total score of ESVADOPA obtained, both in the latent and active phases (dependent variables).

Once the data was analysed after applying the chi-square contrast, a Yates continuity correction was performed, given that the expected frequency of at least one value is lower than 5 (Cramer's V). The level of statistical significance was established at *p* < 0.05.

Finally, two multiple linear regression models were performed using the forced entry method (other methods were ruled out due to the nature of the data obtained) to evaluate the magnitude of the association between the variables such as cultural identity, origin, language barrier, companionship and relationship to the companion with the expression of pain, both in the latent and active phases of labour. The value of the beta coefficient, standard error, *p*-value (as an indication of the variables that should be included in the regression model and the 95% confidence intervals) were calculated.

All analyses were performed using the Statistical Package for the Social Sciences (SPSS) program, version 26, (IBM, New York, NY, USA, for Mac).

## Results

Table 1 shows the socio-demographic characteristics of the parturients, as well as data on accompaniment and oxytocin administration.

**Table 1:** Sample description

Variables	Average (S.D)
**Age**	29.94 (6.8)
**Origin** **n (%)**	
*European*	68 (27.6)
*Bereber*	169 (68.7)
*Sub-shaharan*	6 (2.4)
*Latinoamerican*	3 (1.2)
**Cultural identity**	
*Christian*	59 (24)
*Muslim*	176 (71.5)
*Hebrew*	3 (1.2)
*Atheist*	8 (3.3)
**Language barrier**	
*Yes*	144 (58.5)
*No*	102 (41.5)
**Support during labor**	
Yes	202 (82.1)
No	44 (17.9)
**Relationship with the companion**	
*Couple*	70 (28.5)
*Mother*	23 (9.3)
*Sister*	41 (16.7)
*Mother in law*	17 (6.9)
*Friend*	33 (13.4)
*Other female family member*	18 (7.3)
**Oxytocin administration**	
*Yes*	221 (89.8)
*No*	25 (10.2)

*S.D.* Standard Deviation

Table 2 shows the results of the bivariate analysis for the dimensions and the total ESVADOPA, both in latent and active phases, depending on the women’s cultural identity. In the latent phase we ascertained significant differences between cultural identity and body response (*p* = 0.020; Cramer's V = 0.163), as well as verbal response (*p* = 0.028; Cramer's V = 0.159), with parturient women of Muslim identity showing higher scores in these items. The same is true for the active phase of labour, where Muslim-identified parturient women had the highest scores on all items, with significant differences being obtained for all dimensions of the scale: facial muscles (*p* = 0.013; Cramer's V = 0. 181), body response (*p* = 0.001; Cramer's V = 0.211), verbal response (*p* = 0.000; Cramer's V = 0.215), restlessness (*p* = 0.000; Cramer's V = 0.301), ability to relax (*p* = 0.000; Cramer's V = 0.262) and vegetative symptoms (*p* = 0.045; Cramer's V = 0.153).

**Table 2:** Cultural differences in the expression of pain during latent and active phase during labor process

	**LATENT PHASE**				***p***	**V of Cramer**	**ACTIVE PHASE**				***p***	**V de Cramer**
	**Christian**	**Muslim**	**Hebrew**	**Atheist**			**Christian**	**Muslim**	**Hebrew**	**Atheist**		
**Facial musculature**					0.849	0.081						
**0**	13 (22)	33 (18.8)	0	1(12.5)			0	0	0	0	**0.013**	0.181
**1**	32(54.2)	84(47.7)	1(33.3)	4(50)			24(40.7)	44(25)	0	4(50)		
**2**	14(23.7)	57(32.4)	2(66.7)	3(37.5)			33(55.9)	92(52.3)	2(66.7)	3(37.5)		
**3**	0	2(1.1)	0	0			2(3.4)	40(22.7)	1(33)	1(12.5)		
**Corporal response**					**0.020**	0.163					**0.001**	0.211
**0**	12(20.3)	18(10.2)	0	2(25)			0	0	0	0		
**1**	39(66.1)	88(50)	3(100)	5(62.5)			21(35.6)	26(14.8)	1(33.3)	3(37.5)		
**2**	8(13.6)	68(38.6)	0	1(12.5)			35(59.3)	102(58)	2(66.7)	4(50)		
**3**	0	2(1.1)	0	0			3(5.1)	48(27.3)	0	1(12.5)		
**Verbal response**					**0.028**	0.159					**0.000**	0.215
**0**	20(33.9)	33(18.8)	2(66.7)	2(25)			1(1.7)	1(0.6)	0	0		
**1**	33(55.9)	81(46)	1(33.3)	4(50)			17(28.8)	21(11.9)	2(66.7)	3(37.5)		
**2**	6(10.2)	61(34.7)	0	2(25)			35(59.3)	74(42)	0	3(37.5)		
**3**	0	1(0.6)	0	0			6(10.2)	80(45.5)	1(33.3)	2(25)		
**Restlessness**					0.329	0.118					**0.000**	0.301
**0**	13(22)	26(14.8)	2(66.7)	2(25)			0	0	0	0		
**1**	34(57.6)	96(54.5)	0	5(62.5)			17(28.8)	10(5.7)	1(33.3)	3(37.5)		
**2**	11(18.6)	49(27.8)	1(33)	1(12.5)			39(66.1)	94(53.4)	1(33.3)	4(50)		
**3**	1(1.7)	5(2.8)	0	0			3(5.1)	72(4.9)	1(33.3)	1(12.5)		
**Ability to relax**					0.621	0.098					**0.000**	0.262
**0**	12(20.3)	16(9.1)	0	1(12.5)			0	0	0	0		
**1**	35(59.3)	115(65.3)	2(66.7)	6(75)			11(18.6)	18(10.2)	1(33.3)	1(12.5)		
**2**	12(20.3)	43(24.4)	1(33.3)	1(12.5)			43(72.9)	71(40.3)	2(66.7)	4(50)		
**3**	0	2(1.1)	0	0			5(8.5)	87(49.4)	0	3(37.5)		
**Vegetative Symptoms**					0.636	0.094					**0.045**	0.153
**0**	26(44.1)	72(40.9)	0	4(50)			0	6(3.4)	0	0		
**1**	27(45.8)	87(49.4)	3(100)	4(50)			45(76.3)	109(61.9)	0	6(75)		
**2**	6(10.2)	17(9.7)	0	0			14(23.7)	50(28.4)	2(66.7)	1(12.5)		
**3**	0	0	0	0			0	11(6.3)	1(33.3)	1(12.5)		
**TOTAL ESVADOPA SCORE**	5.37 (2.212)	6.69 (2.302)	6 (1)	5.5 (2.568)	0.887		10 (1.509)	12.57 (2.113)	11.67 (3.786)	10.63 (2.066)	**0.027**	

*p* P value, *ESVADOPA* Scale of pain expression during childbirth

Depending on cultural identity, significant differences were found between groups in the active phase in the total ESVADOPA score, (*p* = 0.027), with Muslim women showing the greatest expression of pain (12.57 ± 2.113), followed by Jewish (11.67 ± 3.786), atheist (10.63 ± 2.066) and Christian (10 ± 1.509) women.

Table 3 shows the final model with two predictor variables of the expression of labour pain in the latent phase. Women who were not accompanied by a companion of choice during childbirth showed greater expression of pain during the latent phase of labour.

**Table 3:** Labor pain associated factors during latent phase

Variables	β	SD	*p*	95% CI	
Support during labor	1.483	0.519	0.005	0.459	2.506
Relationship with the companion	0.238	0.107	0.026	0.029	0.448

*p* P value, *SD* Standard Deviation, *β* Beta coefficient, *CI* Interval of confidence, *F* 3.588, *p* = 0.004

Table 4 shows the final model that includes the three predictor variables of the expression of labour pain in the active phase. Women who were not accompanied expressed greater pain than those who were. During the active phase of labour, women of Berber origin had a higher score on the scale than the other origins. If we consider the language barrier, those women who had greater difficulty expressing themselves with healthcare professionals, expressed greater pain during the active phase.

**Table 4:** Labor pain associated factors during active phase

Variables	β	SD	*p*	95% CI	
Support during labor	1.888	0.459	0.000	0.984	2.791
Origin	0.728	0.239	0.003	0.258	1.198
Language barriers	0.738	0.298	0.014	0.150	1.326

*p* P value, *SD* Standard Deviation, *β* Beta coefficient, *CI* Interval of confidence, *F* 14.914, *p* 0.000

## Discussion

The aim of this study was to evaluate the expression of pain during labour in women that were treated in a hospital of a Spanish border city as well as the associated factors that can influence the expression of this pain.

This study shows a significant relationship between the expression of labour pain and the cultural identity of the women in labour, in addition to other predictive factors of pain, both in the latent and active phases of labour, therefore increasing knowledge in this area. As has been widely demonstrated, there are several factors that can influence the sensory perception of labour pain in addition to neurophysiological and hormonal factors. These, in turn, are modulated by other social, cultural and psychological factors that will influence the way that women externalise this painful experience [24, 25]. Hence, the cultural identity of women in labour influences both the manifestation and the experience of labour pain [16]. In our study, the influence of culture increased the expression of pain specifically as labour progressed and, in more advanced stages the cultural influence is even greater since we can observe that all the dimensions studied are influenced by culture.

Women who self-identified as Muslims had higher scores of pain expression both in the latent and active phases of labour, as compared to women who self-identified as Christians, data that coincide with those reported in other studies [26–29].

People from cultures that value stoicism tend to avoid externalising their pain with moans or screams. They also attempt to keep their faces expressionless, trying to not show any pain even by grimacing, since they think that it will be perceived as weakness if they admit to or show any kind of pain, and may even deny pain when asked. In contrast, other cultural groups are more comfortable with openly expressing their pain, since they seem to cope better and/or may feel alleviated of pain using moans or screams [27]. Similar data were reported by Suárez and Plaza del Pino in which women from Eastern Europe and sub-Saharan women repressed painful expressions, while women of Berber origin tended to be more expressive [16].

There are cultural differences in expressing the anguish of pain in both verbal and non-verbal language. Although pain during labour and delivery is expected by women in all societies, it can be interpreted, perceived, and expressed differently [30].

Our predictive model of the latent phase of labour demonstrated the importance of companionship during labour in addition to the relationship that women had with the companion. In Bohren's review, the importance of the companionship of the women during labour by their partner or a person chosen by her was demonstrated, since they supported the women by providing information about labour, overcoming communication gaps between healthcare professionals and the women in labour and providing non-pharmacological pain relief [31]. According to Power et al. [25], when women were accompanied by a companion of choice, expression of pain was minimised and had a positive impact on coping with the labour experience. This finding is endorsed by the recommendations published by the WHO in 2018 that included the need to offer women in labour the possibility to choose the person who will provide emotional support, therefore encouraging the positive experience of childbirth [32].

In contrast, during the active phase of labour, in addition to the companionship, origin and language barrier come into to play. Moreover, women of Berber origin express greater pain as compared to women of other origins. In this study, the majority of Berber women are practitioners of the Islamic religion, whose conception of pain expression is much greater, as supported by other articles, in which Muslim women expressed and verbalised pain by screaming and crying [16, 26, 27, 33]. According to Yadollahi^32^, cultural factors, such as religious and spiritual perceptions, can determine the reaction to labour-associated pain.

In our study, the language barrier proved to be another parameter associated with greater expression of pain, especially in the active phase of labour. These results are in line with the data obtained by Razzum et al. [34] who carried out a study in Germany with women of different origins. In their study, language barrier was also associated with lower control over pain and its greater expression as compared to women who had no language barrier.

Mustajoki et al. [35] concluded that poor command of language makes it extremely difficult for patients to express their pain specifically, which may lead to an underestimate of the severity of pain and insufficient treatment.

Moreover, the existence of language barriers may lead to a greater vulnerability for the care of these women in labour [36]. This situation could be avoided by transcultural education, at least of the most widely spoken language of the population treated in the specific regions. It has been observed that a greater cultural distance between the woman in labour and midwife increases the possibility of misinterpretation of the expression of pain experienced in this situation [25]. Toledo et al. [37] demonstrated disparities in labour pain management among women with a limited command of English.

Multiple agencies in the United States have included strategies to effectively eliminate racial and ethnic disparities in health care, developing appropriate interventions that include cultural and language characteristics with the aim of reducing inequalities in pain management [38]. Furthermore, the National Partnership for Maternal Safety in the United States recommends that healthcare professionals should address the limitations caused by the existence of language barriers to reduce peripartum disparities by encouraging shared decision-making [39]. In Spain, the high number of migratory movements is giving rise to a change in the dynamics of work. The dizzying change in the manner of childbirth assistance makes us consider the need to put our own constructs to one side and be more open to the knowledge of the new cultures that are increasingly attending the maternity services. Midwives must be trained to assist women from different cultures and should, therefore, be educated in cultural skills that enable them to adapt their daily work to the needs of women in labour. With this in mind, Spanish public administrations should be urged to train health workers, as, to date, only universities have introduced cross-cultural training in their curricula [40–43].

### Limitations

This study’s main limitation was the small number of two of the cultural groups, Jews and atheists. Although it is difficult to quantify the number of non-believers among the general population, it is to be noted that the number of Jewish women in labour included in this study is a proportionally representative group of this cultural group in the city’s total population, as it is a minority group when compared to the population that are self-reported as Muslims and Christians.

## Conclusions

In our study, factors such as cultural identity, origin and companionship during labour affected the expression of pain of women in labour.

In the active phase of labour, a Berber origin, the existence of language barriers and companionship seemed to play an important role in the expression of pain, while in the latent phase of labour support provided by a companion of the woman’s choice proved to be significant in the expression of pain during the process.

There is an evident need for health professionals to be educated and trained in cross-cultural care. Only by doing this will it be possible to offer quality care during delivery to all women, irrespective of their culture, origin or language. Though Spanish healthcare related to the births has high quality levels because the maternal and neonatal morbidity and mortality has decreased, conflicts can appear when the culture of the obstetricians differs from parturients’, appearing moral mistakes in the interpretation of their actitudes. For that reason, the use of tools like ESVADOPA is so important, because it offers to midwives the opportunity of knowing the determinants that have an effect on the expression of labour pains. These tools have to help to health professionals to offer parturients an individualized attention, bearing in mind their individual characteristics. At this way, we can overturn all stereotypes that several times guides the care.

On the other hand, it is important that midwives offer all women the possibility of choosing to be accompanied by a companion of their choice in order to ensure that giving birth would result in a positive experience.

New models of maternity care are necessary that go beyond mere clinical care,, embracing other perspectives such as the culture of the woman in labour.

## Supplementary Information

**Additional file 1.**

## Data Availability

The datasets used and/or analysed during the study are available from the corresponding author upon reasonable request.
