# Supplementary material for: Cultural influence on the expression of labour-associated pain
Source: BMC Pregnancy Childbirth. 2022 Nov 14;22:836. doi: 10.1186/s12884-022-05173-1 (PMC9664611; doi:10.1186/s12884-022-05173-1)
Supplement: Supplementary file 1 — Additional file 1. [file 12884_2022_5173_MOESM1_ESM.docx]

| **Item** | **Sub-Item: Score/Meaning** | **Score** |
| --- | --- | --- |
| FACIAL MUSCLES (FM) | 0. Relaxed during the entire contraction |  |
|  | 1. Relaxed with slight facial tightening during most of the contraction |  |
|  | 2. Frowning/grimacing/clenched teeth only during the peak of contraction |  |
|  | 3. Frowning/grimacing/clenched teeth during the entire contraction |  |
| BODY RESPONSE (BR) | 0. Relaxed during the entire contraction |  |
|  | 1. Relaxed with slight contraction of hands, arms, and/or toes and legs during most of the contraction |  |
|  | 2. Increased: flexion of the fingers, arms, and/or toes and legs during the peak of contraction |  |
|  | 3. Increased: flexion of fingers, arms, and/or legs during the entire contraction |  |
| VERBAL RESPONSE (VR) | 0. In silence or fluid conversation during the entire contraction |  |
|  | 1. Mild moans and sobs during most of the contraction |  |
|  | 2. Shouts, complains, grunts, and sobs at the peak of contraction |  |
|  | 3. Shouts, complains, grunts, and sobs during the entire contraction |  |
| RESTLESSNESS (R) | 0. Calm, relaxed, normal movements during the entire contraction |  |
|  | 1. Calm, relaxed, slight movements indicating restlessness during most of the contraction |  |
|  | 2. Occasional movements indicating restlessness and/or changes in position at the peak of contraction |  |
|  | 3. Continuous movements indicating restlessness and/or changes in position during the entire contraction |  |
| ABILITY TO RELAX (AR | 0. Relaxed and calm throughout the contraction |  |
|  | 1. Relaxes with the touch and/or voice of the companion or health professional |  |
|  | 2. Begins to present difficulties with relaxing with the touch and/or voice of the companion or health professional |  |
|  | 3. Rejects the touch and/or the voice of the companion or health professional |  |
| VEGETATIVE SYMPTOMS (VS) | 0. No vegetative symptoms |  |
|  | 1. Sweating and/or nausea |  |
|  | 2. Sweating, nausea, and/or dizziness |  |
|  | 3. Sweating, nausea, vomiting, dizziness, increased blood pressure (BP), tachycardia, and/or dilated pupils |  |

Total Score:

<1: *Does not express pain*

1–6: *Expresses mild pain*

7–12: *Expresses moderate pain*

13–18: *Expresses intense pain*
